# The window ductus: Circulatory arrest as an option for surgical repair

**DOI:** 10.4103/0974-2069.74047

**Published:** 2010

**Authors:** Vijay Agarwal, Muslim Mustaev

**Affiliations:** Cardiothoracic Surgery Unit, Indus Hospitals, Visakhapatnam, Andhra Pradesh, India

**Keywords:** Aortic operation, cardiopulmonary bypass, congenital heart disease surgery

## Abstract

The window ductus represents a very rare anatomical anomaly. Only a few cases of successful surgical repair have been reported. We present a 12 year old boy in whom window ductus was diagnosed and successfully operated. The operation included closure of the large (2.5 cm×0.5 cm) connection between the main pulmonary artery and the aortic arch using cardiopulmonary bypass and hypothermia with circulatory arrest.

## INTRODUCTION

The window ductus differs from the usual type of patent ductus arteriosus in that the lumen of the pulmonary artery communicates directly with that of the aorta, so that the ductus has no recognisable length, and there is a window where two great vessels lie in apposition at the distal end of pulmonary trunk (Edwards, 1960). The window ductus is diagnostically and surgically challenging, as this case represents.

## CASE REPORT

A 12-year-old boy was referred from the cardiology unit with a diagnosis of large patent ductus arteriosus (PDA). Clinical examination revealed no audible murmur on auscultation of the heart. The chest roentgenogram showed features of severe pulmonary hypertension and the echocardiogram showed presence of a large PDA with a left to right shunt with severe pulmonary hypertension. The PDA was restricted by only 10 mm Hg.

**Figure 1:**
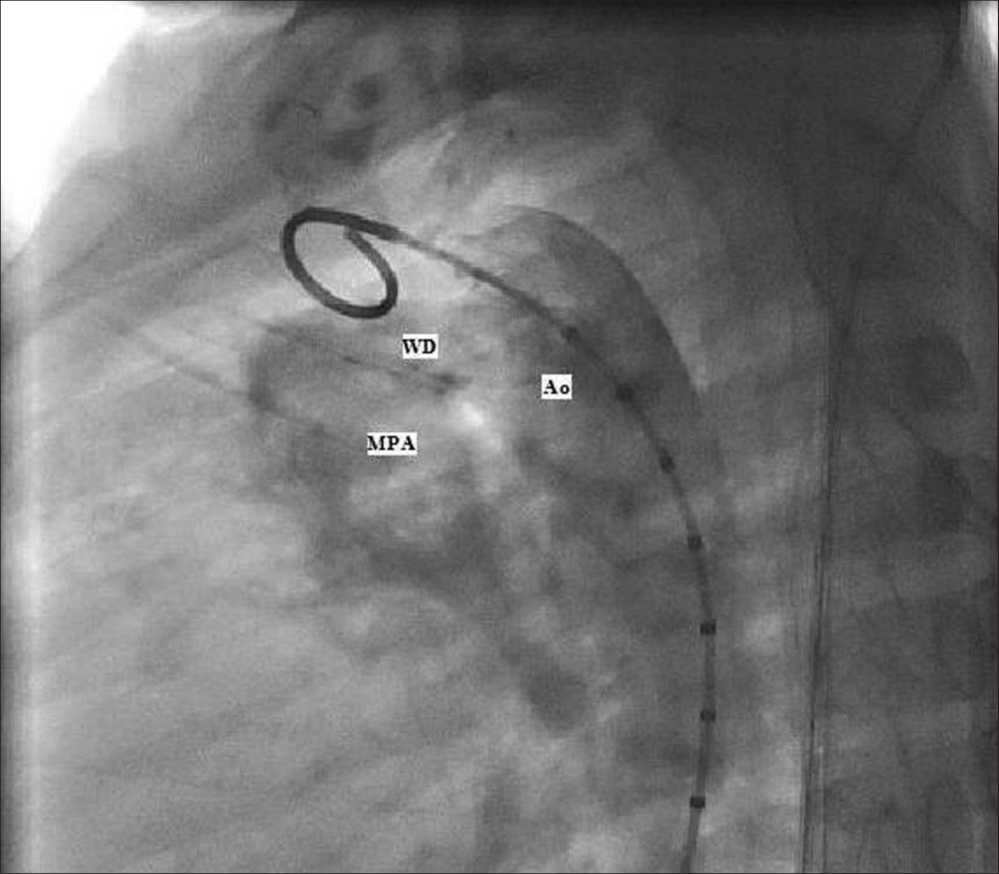
Angiography. MPA, main pulmonary artery; Ao, aorta; WD, window ductus

Cardiac catheterization showed a large left-to-right shunt with pulmonary artery pressure (PAP) of 100/50 mm Hg (mean=75 mm Hg). The aortic pressure measured 100/60 mm Hg (mean=80 mm Hg) [Figure 1] Device closure of this defect was attempted, but was unsuccessful and hence the boy was referred for surgical closure. Pre-operatively, he was treated with Sildenafil (0.5 mg/kg/day) for 10 days.

**Figure 2:**
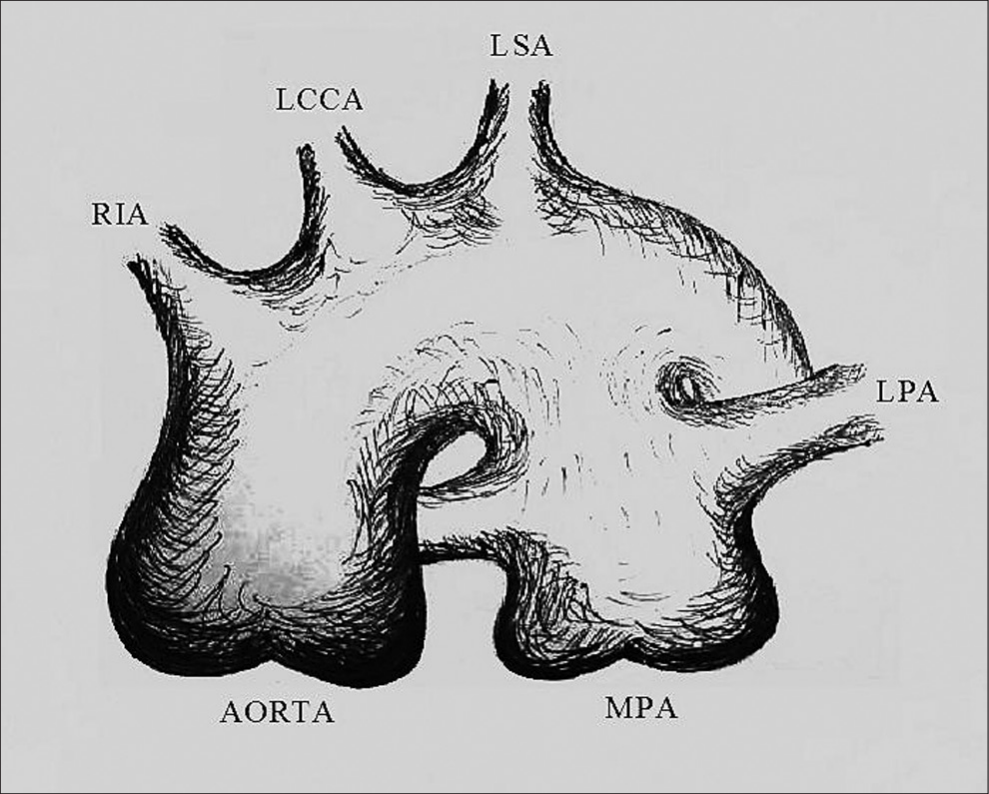
Schematic view of the window ductus. MPA, main pulmonary artery; LPA, left pulmonary artery; RIA, right innominate artery; LCCA, left common carotid artery; LSA, left subclavian artery

The patient was operated through the median sternotomy; the main pulmonary artery (MPA) was very tense and demarcation between the pulmonary artery and the aortic arch was not very evident [Figure 2]. Cardiopulmonary bypass was established using aortobicaval cannulation. The heart was arrested using cold crystalloid antegrade cardioplegia and dissection was continued. It was noticed that there was no length to the ductus. The recurrent laryngeal nerve, which serves as an indicator of classical PDA site was not visible. During the dissection, there was difficulty in maintaining perfusion pressure and attempts to maintain it by clamping the PDA were unsuccessful.

**Figure 3:**
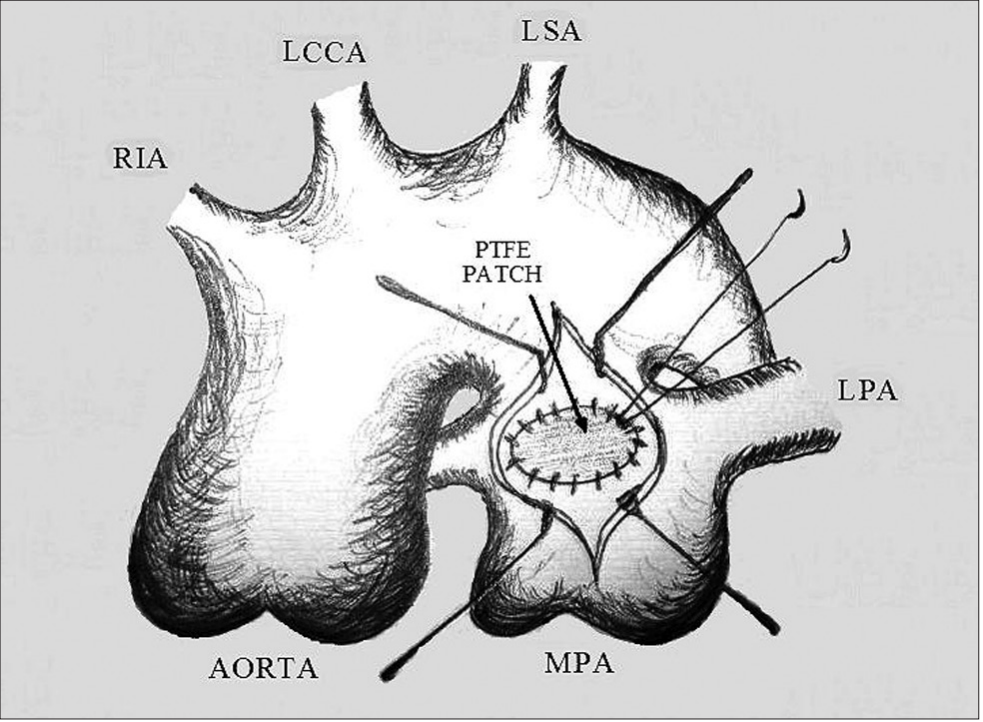
Scheme of the operation. Closure of window ductus by the PTFE patch. MPA, main pulmonary artery; LPA, left pulmonary artery; RIA, right innominate artery; LCCA, left common carotid artery; LSA, left subclavian artery

To prevent distention of the right ventricle (RV), the right atrium and the MPA were incised to open the large aortopulmonary connection. Attempts to occlude the opening with Foleys urinary catheter were unsuccessful. To attain and delineate the opening from the MPA clearly, the patient was cooled to 24°C and circulation was stopped (total circulatory arrest). The aortopulmonary connection was found to be about a 2.5-cm-large oval window with no length and no crumpled tissue classic for PDAs. This was then closed with the polytetrafluoroethylene (PTFE) patch (GORE-TEX, W. L. Gore and Associates Inc.: Delaware) from inside the MPA with polypropylene 5-0 (Ethicon, Inc. New Jersy) continuous sutures [Figure 3]. The total circulatory arrest time was 18 min and the cross-clamp time was 1 hour and 8 minutes The MPA incision was closed by 5-0 polypropylene in two layers of continuous sutures and the flow was restarted. In order to prevent post-operative RV failure, we stretched the patent foramen ovale (approximately 4 mm in diameter). After deairing the heart, the patient was weaned off the cardiopulmonary bypass. Intraoperative monitoring of PA pressure showed systemic PA pressures (90/40 mmHg), but with stable hemodynamics. The chest was closed and the patient was shifted to the intensive care unit (ICU) in a stable condition.

During the operation and in the post operative period, continuous infusion of Milrinone (0.7 mg/kg/h) and Phenoxibenzamine (0.5 mg/kg/h) were administered. Three hours after shifting to the ICU, echocardiogram was performed. It revealed that the post-operative PA pressure had come down dramatically to 50% of the systemic pressure and the patient was extubated after 6 hours. On the 3^rd^ post-operative day, the echocardiogram showed a decrease in PAP to 45/20 mmHg. The patient was discharged on the 7^th^ day after the operation and was advised to continue oral Sildenafil. At the time of discharge, his neurological status was found to be normal.

## DISCUSSION

According to previous reports,[1–3] window ductus (WD) is a very rare and surgically challenging anatomical entity, Our patient who was a 12-year-old boy is the oldest among all reported cases in the world literature. WD has a number of significant anatomical features, including its location (typical for PDA), large size (mostly more than 2 cm) and a characteristic view of the MPA continuing into the aortic arch.[4,5] Internally, no thickened ductal intimal pads are seen. Most authors regard the WD as an atypical PDA and not as an aortopulmonary window due to its very distal location, i.e. after bifurcation of the PA.

Presence of the WD is surgically challenging, both, in terms of planning and execution.

Only one case of successful operation reported in the literature was performed using cardiopulmonary bypass. In that case, the WD was ligated before ventricular septal defect closure.[2] Still, there are no clear recommendations on the surgical strategy toward WD – whether it should be operated with or without cardiopulmonary bypass – and also there is no unanimous opinion regarding use of circulatory arrest.

On one hand, dissection of the short and large vascular communication between the aortic arch and MPA without the instituting cardiopulmonary bypass may cause rupture with dramatic bleeding while, on the other hand, use of cardiopulmonary bypass is unable to maintain adequate perfusion pressure due to the large left-to-right shunt. This can result in significant drop in brain perfusion pressure while separating the communication. Therefore, under these circumstances, circulatory arrest seems to be the most preferred option as was done in this case.

Our case has shown that hypothermia to 24°C is sufficient to provide a reasonable cerebral protection for a short period of time (<20 min). We applied circulatory arrest after external dissection of the MPA and its branches and just prior to starting the the intracardiac repair. This helped in limiting the time of total circulatory arrest. The separation of the MPA from the aortic arch was technically challenging. One of the most important steps was to dissect and snare the MPA branches as it helped in obtaining a dry operative field.

The WD could be closed with a big-sized patch, applying high-flow suction. Additionally, deairing after the closure of the WD had to be carefully performed as there might be air collection in the aortic arch. All the steps needed to be performed precisely and quickly. Dissection and snaring of the neck vessels may be useful in preventing air embolism. Other measures suggested for such technically demanding cases include flushing of the operative field with CO_2_ along with Trendelenburg positioning of the patient.
